# Assessment of outcome after hip fracture: development of a universal assessment system for hip fractures

**DOI:** 10.1051/sicotj/2016018

**Published:** 2016-06-03

**Authors:** Thomas M. Bowers, Martyn J. Parker

**Affiliations:** 1 Royal Gwent Hospital Newport Gwent NP20 2UB UK; 2 Peterborough City Hospital, Edith Cavell Campus Bretton Gate Peterborough Cambridgeshire PE3 9GZ UK

**Keywords:** Hip fracture, Fragility fracture, Scoring, Follow-up, Pain, Functional outcome, Social dependency

## Abstract

*Background*: The aim of the study was to refine current evaluation systems used to assess outcome after a hip fracture and to devise a simple and practical system to assess all hip fracture patients.

*Methods:* Three continuous scales were defined for pain, mobility and functional independence. These were all found to have an acceptable degree of inter-observer agreement. The pre-fracture mobility and independence scores were related to the one-year mortality for a consecutive series of 381 patients.

*Results:* Scores for mobility and functional independence were highly predictive of mortality (*p* < 0.0001).

*Conclusions:* It is recommended that the outcome after hip fracture should be standardised to these principle outcomes of pain, regain of mobility and independence and mortality. These scores can be use to assess progress and identify those who may require additional assessment or intervention.

## Introduction

Various scoring systems are available for the assessment of hip function. Unfortunately most of these are not suitable for use in patients presenting with hip fracture [1]. Those who sustain hip fractures are not always fit and well in their injury state [2]. They may already require assistance at home with personal care or reside in institutional care prior to admission. In particular, a significant proportion of patients suffer with a degree of cognitive impairment [3, 4]. Failure to allow for such groups within a scoring system limits any potential role in research and audit.

Performing repeated clinical assessments within this cohort can be problematic. The frail elderly may be unable to attend clinics without the provision of hospital transport or additional carers. Postal questionnaires are an available option, although obtaining responses can be challenging, particularly in patients with cognitive impairment [5, 6]. A system that can be used readily via telephone, either on its own or to support a postal survey, is highly desirable [7, 8].

The aim of this study was to review and refine previous assessment systems to develop a simple and reliable assessment method that can be used for all hip fracture patients.

## Patients and methods

We reviewed commonly used assessment and evaluation methods that had been in common use for hip fracture patients. From these we identified three principal components: pain at the hip, walking ability and functional independence relating to daily care needs. The aim was then to formulate a linear scale for these outcomes. Our system was based on using higher scores to represent a poorer degree of mobility, increased social dependence and increased pain in the hip. All these were based on the previous evaluation scores.

The mobility scale (Table 1) was modified from that of previous scores [9, 10]. A score of one represents a patient who has no need for any walking aid and has no restriction in walking distance, through to 10, which represents a bedbound individual.

**Table 1.** Mobility scale.

Score	Mobility
1	Never uses any walking aid and no restriction in walking distance.
2	Never uses any walking aid, but walking distance limited to less than one kilometre.
3	Occasionally uses a walking aid when out walking.
4	Normally uses one walking stick or needs to hold on to furniture.
5	Normally uses two sticks or crutches.
6	Mobilises with a frame alone, without the need for assistance.
7	Mobilises with a frame and the assistance of one other person.
8	Mobilises with a frame and the assistance of two people.
9	Bed to chair (with or without assistance), or wheelchairbound.
10	Bedbound most or all of the day.

Social dependency (Table 2) was based on previous scales, with Activities of Daily Living (ADL) given a value of one for a person who is completely independent and living at home not requiring any support or assistance, to eight which is a fully dependant requiring patient requiring care in hospital [11]. The ADL considered include the basic activities of washing, dressing, toileting and eating. We considered more advanced ADL to be housework, cooking and shopping (these activities require planning, stepwise execution and a potentially greater level of physical effort).

**Table 2.** Social dependence scale.

Score	Social dependence
1	Completely independent. Requires no assistance in basic or advanced activities of daily living (ADL) including shopping.
2	Minimal assistance. Requires occasional help up to twice a week from family, friends or other services with some activities such as shopping or gardening.
3	Moderate assistance. Requires regular assistance more than twice a week but less than seven times a week with some ADL such as bathing, washing or heavy housework.
4	Regular assistance. Requires daily help to assist with ADL.
5	Dependent. Requires regular help more than once a day with many basic ADL such as preparing food and housework but remains living at home.
6	Severely dependent. Living in residential care. Full-time care facility but independent of at least one basic ADL such as being able to dress or go to the toilet without help.
7	Fully dependent. Living in nursing home, skilled nursing home or long-term hospital facility with full-time nursing care. Patient requires assistance in most ADL living such as washing, dressing and getting to the toilet.
8	Patient temporarily resident in hospital requiring both nursing and medial care.

The pain scale we have used is modified from that of Charnley to specifically reflect pain originating from the hip (Table 3) [12]. A score of zero is given if the patient or their attendant carer is unable to answer, else the pain is rated one (no pain in the hip) to eight (constant and severe pain requiring regular strong analgesia).

**Table 3.** Pain scale.

Score	Pain
0	Unable to answer.
1	No pain at all in the hip.
2	Occasional and slight pain. May occasionally take mild analgesia such as paracetamol.
3	Some pain when starting to walk, no rest pain. Occasional analgesia taken.
4	None or minimal pain at rest, some pain with activities, frequent mild analgesia.
5	Regular pain with activities which limits walking distance. Occasional or mild rest pain.
6	Frequent rest pain and pain at night. Pain on walking. Regular mild analgesia and occasional stronger analgesia taken.
7	Constant pain present around the hip. Regular mild analgesia and frequent strong analgesia.
8	Constant and severe pain in the hip requiring regular strong analgesia such as opiates.

To evaluate the reliability of these scores, 50 patients, who had previously sustained hip fracture, were assessed at their follow-up appointments by two orthopaedic clinicians (one Consultant and one Specialist Orthopaedic Resident). Patients were seen independently without conferring and all results collected independently until the end of the study. Patients were assessed on an “as seen” basis. This, as much as possible, allowed a representative sample of patients with hip fracture in our local population, including those with cognitive impairment. We did not select patients based on either fracture pattern or treatment method.

To assess the ability of the mobility and independence scores to predict mortality at one year from injury, a further 381 consecutive hip fracture patients were admitted to one unit. All patients were assessed at one year from injury to determine mortality. Three patients could not be contacted and enquiry was made to the Office of Population Censuses to check whether these patients had not died. The mean age of these patients was 80.5 years (range 39–105 years) and 274 (72%) were female. At the time of injury 288 (76%) were admitted from their own home, 57 (15%) from residential care, 19 (5%) from nursing homes, 16 (4%) sustained a fall while acutely admitted in hospital. All these patients were scored on admission using the new mobility and social dependency scores by a single observer. The assessment was for their score at the time just before the injury.

### Statistical analysis

The intra-observer reliability between the two clinicians was measured using a Weighted Cohen’s Kappa test, performed using a desktop statistics package. Kappa is a coefficient of agreement which has a value varying from +1 representing perfect agreement, through zero representing an agreement no better than chance, to −1, absolute disagreement. There is no definite definition of level of agreement which is acceptable but values of 0.75 are felt to represent excellent agreement, 0.75–0.5 is good and less than 0.5 is poor agreement [13]. The mean scores for those patients who died were compared to the mean scores for the survivors by *t*-test.

## Results

### Mobility, social dependence and pain scoring

The mean scores and standard deviation for the three domains assessed by each rater are summarised in Table 4.

**Table 4.** Mean scores and standard deviation.

Scale	Observer 1 Mean	Observer 2 Mean
Mobility	5.14, *SD* 2.34	4.8, *SD* 2.17
Social dependence	3.58, *SD* 2.16	3.68, *SD* 2.12
Pain	2.8, *SD* 1.80	2.46, *SD* 1.39

### Inter-observer reliability

In the assessment of mobility and social dependency, excellent inter-observer correlation was demonstrated with kappa values of 0.77 and 0.82 (Table 5). The pain score showed a lower kappa value of 0.5 representing good agreement.

**Table 5.** Inter-observer agreement.

Scale	Kappa
Mobility	0.77
Social dependence	0.82
Pain	0.50

### Mortality prediction at one year

Eighty-six (29%) of the consecutive series of 381 patients had died at one year from injury. The mean mobility scores for those patients who died were 4.5 versus 3.2 for those who were alive at one year (*p* < 0.0001). The mean social dependency score was 4.9 for those who had died, versus 3.2 for those who were alive at one year (*p* < 0.0001).

## Discussion

There is a need for a universally accepted assessment method to define the outcome after a hip fracture [1]. The most important outcome measures are mortality, residual pain, regain of mobility and regain of independence. Any assessment system must be suitable for use for all types of patients including those in institutional care and those with dementia. Because of the fragility of some patients the assessment may need to be carried out over the phone if the patient cannot attend the outpatient review and it must be easy to complete with an acceptable degree of intra-observer variation. The assessment system described here satisfied all these criteria. In addition, the linear scales used allow the patient progress to be monitored.

In addition, any assessment of function must take into account the patient’s pre-fracture status. The traditional scoring systems such as the Harris Hip Score [14], described for use on young patients with acetabular fractures, assume normal pre-injury hip function. This is clearly incorrect and inappropriate for the hip fracture population. For example, a patient who before breaking their hip walked with a Zimmer frame and at follow-up had identical mobility with a pain-free hip would be graded as a “fair” outcome out of a possible range of excellent, good, fair and poor. Some studies have tried to correct for this by subtracting from the Harris Hip Score an assessment of the pre-fracture walking ability, but this results in the pain assessment becoming the predominate part of the score. We have to strongly support previous work that stated summating different outcomes into a single score is illogical and not appropriate for this group of patients [15, 16].

The scoring system we have employed in this case produced similar mean scores for all three domains, which when further analysed using a Weighted Cohen’s kappa test demonstrated excellent correlation between both clinicians when assessing Mobility and Social Dependence. Significantly, in the one-year data, we demonstrated that higher scores in these domains were independently associated with a greater risk of mortality. This is in line with the previously published scoring system, which conferred a similarly high level of risk to patients with poorer mobility and increased social dependence.

Pain scoring proved to be problematic. Although there was a moderate agreement between both observers in the study, it was not to the same degree as that seen in assessment of mobility and social dependence. This may reflect a degree of subjectivity in perception of a patient’s pain by both the patient and the clinician. It is easy to observe a patient’s mobility and what walking aids they may require and similarly, what social support they are requiring to meet a satisfactory standard of personal care, but less clear as to how pain affects a patient. Pain perception and tolerance is highly multifactorial and varies between individuals and indeed in the same individual depending on a variety of confounding factors [17]. Therefore, it is unrealistic to expect the pain score to show a strong degree of inter-observer correlation.

The assessment system suggested here can be readily employed at the time of admission by the admitting orthopaedic clinician as part of their standard clerking, or at a suitable time in the peri-admission period by another orthopaedic practitioner [18]. Scoring can then be repeated in the follow-up period and return to function noted in a linear fashion. As an extension of this, it may be possible as a result of repeated assessments to identify patients whose progression is slow and target them for enhanced rehabilitation measures or additional orthopaedic assessment to check on the hip surgery.

Many previous assessment systems also collect information on limb length discrepancy, rotation and range of motion. This data requires the patient to be able to attend for and be compliant with the examination, which is not always the case in this patient group. Additionally, it is worth considering that the contralateral limb may not be “normal” to allow direct comparison, it is estimated that, in this cohort of patients, 9% will have had previous hip fracture or elective hip surgery [19].

In summary, the pain, mobility and social dependency scores both demonstrate good to excellent intra-observer variability and a significant predictive value in mortality at a year post-injury. By assessing these domains independently, we can develop a more accurate clinical picture of our patients and focus on specific issues, such as provision of appropriate mobility aids and prescription of sufficient analgesia to maintain quality of life.

This demonstrates the extent to which often the most simple, straightforward questions a clinician will ask their patient or those caring for them can be the most vital indicators of progression following injury and surgical treatment. These can be used as a standalone measure to observe a linear progression of functional regain and mortality prediction, as well as being used in research and audit studies.

## Conflict of interest

MP has no conflict of interest directly in relation to this work. He has received expenses and honoraria from a number of commercial companies and organisations for giving lectures on different aspects of hip fracture treatment. In addition, he has received royalties from B. Brawn Ltd related to the design and development of an implant used for the internal fixation of intracapsular hip fractures.
